# Epidemiology and Clinical Management of Early Childhood Caries in Israel

**DOI:** 10.3389/fpubh.2019.00280

**Published:** 2019-10-09

**Authors:** Aviv Shmueli, Moti Moskovitz, Elinor Halperson, Diana Ram, Avia Fux-Noy

**Affiliations:** Hebrew University-Hadassah School of Dental Medicine, Jerusalem, Israel

**Keywords:** caries, prevalence, early childhood, prevention, Israel

## Abstract

**Aim:** The aim of the present review is to describe the current status of early childhood caries (ECC) in Israel in aspects of epidemiology, prevention, and management.

**Methods:** PubMed search was performed using the words caries, children, Israel. Demographic data was collected from the Israeli Central Bureau of Statistics.

**Results:** The decayed, missing, and filled teeth index was 2.72 in 1992 and 2.56 in 2016. The proportion of restored teeth has increased. A number of preventive programs are ongoing but a general preventive program is lacking at the national level. From 2010, every child in Israel is eligible to receive free dental treatment.

**Conclusion:** The gaps in understanding of the epidemiological profile of ECC in Israel is a call for more research conduct on ECC in the country.

## Introduction

Israel is a relatively small country with a population of almost 9 million people, located in the Middle East. The population is diverse with about 6.6 million Jews and 1.9 million Arabs. Since the establishment of the State of Israel in 1948, Jewish persons from around the world have immigrated to the country. According to the published registry, Israel's population at the end of 2016 was 8.63 million people of which 2.8 million (aged 0 to 17 years) are children. This is about 33% of the population of which 71.3% are Jews, 25.7% are Arabs (Muslims, Christians, and Druze), and 3% are of other backgrounds (1). Children under age 14 years comprise 28.2% of the population (1). This item is high compares to the population distribution in western countries but relatively low in comparison to Israel's neighboring countries. Percentage of children is higher in the northern and southern parts of Israel. Places where children constitute more than half of the population typically belong to religious population (1).

Israel's relatively small population comprises diverse ethnic groups. These subpopulations have specific socio-demographic and cultural characteristics that may affect the tendency to develop caries in general and early childhood caries in particular. There are some population groups for example the ultra-orthodox Jews that are characterized by large families (more than 4 children per family). In these communities the dental awareness is sometimes low, and many children are referred to dental treatment under sedation or general anesthesia due to their young age and complexity of treatment.

In 1981, water fluoridation was started in Israel. The first fluoridation station was in the capital city, Jerusalem. Despite the significant decline in caries prevalence and severity, which was attributed to water fluoridation, and despite the recommendations of public health experts, the Israeli Minister of Health decided to discontinue public water fluoridation in 2014. The reasons to this step were concerns about the influence of fluoride on general health. Israel is located in the hot region of the Middle East and there is substantial shortage of natural water. Most of the water (about 80%) that will be provided in the coming years will be from water desalination. The implication is that most of the water in Israel will not be fluoridated.

Public debates and discussions are ongoing regarding water fluoridation. The dental professional advisors recommended the minister of health to add fluoride to the drinking water, the current minister of health is willing to do so, hopefully, the problem will be resolved soon, in favor of the dental health of Israeli children (2). The current review describes the status of ECC in Israel, prevention and treatment modalities.

## Epidemiology of Early Childhood Caries (ECC)

There are only a few surveys on ECC conducted in Israel. This is due to difficulties such as the lack of accessibility to a large representative sample of children younger than 5 years, since the compulsory school enrolment begins at this age (“Israeli compulsory education”)^1^ Examining very young children in Mother-and-Child Health Centers raises biases because this population does not necessarily reflect the general population. To absence of a national survey on ECC contributes to the challenges with planning for prevention and early detection of this disease.

The findings of a national survey of 5-year-old children were published in 1992 (3). The sample of 767 children were recruited from 25 kindergartens around the country and examined using World Health Organization (WHO) criteria. No radiographs were taken. The mean decayed, missing and filled teeth (dmft) were 2.72 for children aged 5 to 6 years, and the prevalence of caries was 58.7%. No differences were found between genders or between urban and rural populations (3).

Another survey was performed in Ashkelon, a peripheral but large city (above 100,000 residents) in southern Israel (4). Of the 182 5-year-old children examined in that survey, 57% had caries. The mean dmf (t) was 2.08 + 2.64. An interesting finding of this survey was that the f component of the dmft was 0, which means that these children were not treated (4). Children at the lower end of the socio-economic scale showed high rates of severe early childhood caries. Populations that are predominantly low on the socio-economic scale are Bedouins, Ultra-Orthodox Jews, refugees from Africa and immigrants. These populations are characterized by low income and a high number of children. According to the Israeli national insurance institute, 463,300, families in Israeli were of low socio—economic level in 2016, among them 842,300 children (5). In 2007, a survey among 5-year-olds in 28 of 70 local authorities providing school dental services reported that 64.7% of children had tooth decay, with an average dmft of 3.31 (6).

Livny et al. (7), by using visual caries detection method, examined 102 Bedouin children for caries, using the World Health Organization (WHO) (1997) criteria. The children were aged 12 to 36 months from the Jahalin tribe, a previously nomadic tribe dwelling on the eastern outskirts of Jerusalem. White spot lesions were not recorded as caries. The investigators reported severe early childhood caries in 17.6% of the children. The prevalence of early childhood caries was significantly associated with older children's age (25 to 36 months), larger family size, and mother's lower education level and poorer dental appearance. The average number of children per family in that study was 4.6 and the maximum was 13. The researchers assumed that in a large family, it is more difficult for the parents to provide optimal healthcare according to each child's needs, including healthy feeding and oral hygiene practice (8).

In attempting to understand the prevalence trends of caries in Israel, and specifically ECC, Sgan-Cohen et al. (9) compared average defs (decayed extracted filled surfaces) and dmfs among first-grade children in south-west Jerusalem. According to municipality records, the neighborhoods surveyed represent the lower middle class. The examinations were conducted in 1983, 1992, and 2005, in accordance with the 1977 WHO's recommendations. Data indicated a consistent decline in disease severity: a reduction in the defs from 13.95 to 8.09 and 5.07 in 1983, 1992, and 2005, respectively.

In 2016, Natapov et al. (10) published a survey of 6 year-old-children residing in 23 local authorities that were randomly selected nationwide. Although Ecc is defined as caries before age six. Trends can be learned from this data about the caries experience of this children in their early years of life. The children were examined according to the WHO Oral Health Survey Methods 4th edition protocol. Of 1,210 children examined, 61.7% had dental decay and only 38.3% were caries free. The mean dmft was 2.56: d−1.41 (teeth with untreated caries), f = 1.15 (teeth damaged by decay and restored); no teeth were missing due to caries. The prevalence of dental caries was rather consistent, an average of over 2 teeth were affected per child. In this survey, the f component was higher than previously reported, especially in the Jewish sector, yet still lower in the Arab sector.

## Early Childhood Caries Prevention Programmes

### The Impact of Free Access to Toothbrush and Toothpastes on Early Childhood Caries Incidence

A study was conducted to assess the impact of access to free toothbrushes and toothpastes on the incidence of ECC in 1,500 6-month-old infants recruited from Mother-and-Child Health Centers over a 2-year period. At age 2.5 years, 596 of the 1,500 children were examined. The early childhood caries prevalence was 15.3%. No difference in caries prevalence was found between children whose parents received the study intervention and those whose parents did not. The proportion of children who brushed twice daily was 13.9%, while 26.8% of the children did not brush at all. Eighty-one percent of the parents reported that their children went to bed at night with a bottle. Among children who drank sugar-sweetened beverages, the prevalence of early childhood caries was significantly higher than among those who drank milk or natural juice (18.8 vs. 8.9%). The study highlighted that tooth brushing might not be the only nor optimal public health response for early childhood caries in Israel (8).

### Community Health Education, Bottle-Feeding Practices and Tooth Brushing Behavior of Infants in Jerusalem

In this quasi-experimental study designed to compare the impact of a structured health education programme on infant's bottle feeding and tooth brushing behavior, parents of 727 children aged 6 to 12 months from Mother-and-Child Health Centers in Jerusalem were recruited (8). Parents in the intervention group received structured health education information over the telephone, and half were randomized to receive fluoridated toothpaste and tooth brushes. The control group received did not receive an organized educational intervention. Six months into the study, parents in the control group reported a 32.5% increase in tooth brushing for infants, compared to baseline. Tooth brushing frequency increased by 45.1% for infants of parents who received toothpaste and toothbrushes, by 43.7% for infants whose parents received health education information only, and by 60.4% for infants whose parents received health education, toothpaste, and toothbrushes (*P* = 0.0002) (8). Modification of bottle-drinking practices in this programme was unsuccessful. The study therefore recommended that free distribution of toothpaste and toothbrushes, in conjunction with a structured oral health education programme to parents of infants, is effective in promoting good oral hygiene practices (11).

### The Impact of a Prenatal Infant Oral Health Programme Implemented Through Baby Clinics

In 2003, members of the Pediatric Dentistry Department of the Hadassah School of Dental Medicine initiated a Baby Clinic in Jerusalem, based on the Brazilian concept of prenatal prevention. The objective of the project was to describe the implementation and outcome of a prenatal infant oral health programme that focused on pre- and post-natal oral health education for parents, and caries prevention in their first-born babies. The intervention commenced in late pregnancy and continued until the child reached age 3 years. Twenty-four, 30-min long prenatal power-point dental education lectures were delivered to 300 young, first-time parents to be, from the 8 to 9th month of pregnancy. Participants attended the “Course for Birth Preparation” at the Hadassah Hospital, Jerusalem. Lectures were given by pediatric dentists. The lectures emphasized the relation between the mother's oral health and the oral health of the expected baby, and provided recommendations about feeding and oral habits for the expected baby. Parents were invited to bring their babies for the first visit when their first tooth erupted or between ages 6 and 12 months. A brochure containing a summary of the information from the lecture was distributed at the first or second follow-up visit, to provide parents the opportunity to review the information at home and share it with other family members or friends who did not attend the lectures.

At each recall appointment, the young parents received anticipatory guidance for prevention of dental disease, and brushing instructions were demonstrated in the child's mouth, using the “lift the lip technique.” Follow-up visits were recommended according to a protocol, based on individual caries risk assessment: low risk, every 6 months; medium risk, every 4 months; and high-risk babies, every 2 months. For the high-risk group, the teeth at risk were treated with fluoride varnish. A “dental home” was also achieved for the participating families. The intervention significantly reduced the incidence of early childhood caries. By the first visit, 70% of the 137 parents who attended already brushed their children's teeth. The incidence of early childhood caries was 2.9% over the 3 years of the study, a figure significantly lower (*p* < 0.001) than the 15.3% reported by Livny and Sgan-Cohen (12).

### Caries Risk Assessment Tool and Prevention Protocol for Public Health Nurses in Mother-and-Child Health Centers, Israel

In Israel, Mother-and-Child Health Centers provide free preventive services for pregnant women and children by public health nurses. A caries prevention program in health centers started in 2015. Nurses underwent special training regarding caries prevention. A customized Caries Risk Assessment Tool and Prevention Protocol for nurses, based on the tool implemented by the American Academy of Pediatric Dentistry, was introduced. A two-step evaluation was conducted, which included a questionnaire and in-depth phone interviews. Of 46 health centers included in the study, 28 returned a completed questionnaire. Most nurses believed that oral health preventive services should be incorporated into their daily work. In the in-depth phone interviews, nurses stated that the integration of the program into their busy daily schedule was realistic and appropriate. The lack of a specific dental module for managing computerized data was mentioned as an implementation difficulty (13).

## Early Childhood Caries Treatment

### The National Health Insurance Scheme

Pediatric dental service was not part of the national health services until June 2010. Prior to this date, dental services such as dental examination, preventive treatments and pediatric dental treatments were not provided by the Ministry of Health. Access to dental services was limited, especially for underprivileged populations and those living in the peripheral districts of the country. The June 2010 legislation of the National Health Insurance for children under age 8 years affords access, for every preschool child, to preventive and operative care, which was usually provided by general dental practitioners. The service is operated by four health maintenance organizations that provide all the other public medical healthcare services. The service includes the provision of all operative dentistry needs of children in the public healthcare service and operating referral centers. At such centers, very young children are usually treated under conscious sedation or general anesthesia. Specialists in pediatric dentistry generally provide the care (14).

## Conclusion

The prevalence of early childhood caries in Israel has not changed much over the years. According to the latest survey, treated caries are more prevalent in children than in the past but dmft is still relatively high. Particular emphasis on preventive measures are needed, especially in underprivileged populations.

## Author Contributions

All authors have made substantial contributions to the conception and design of the work, the acquisition, analysis, or interpretation of data for the work, and have made substantial contributions to the drafting the work and revising it critically for important intellectual content. All authors approved the final version to be published and agreed to be accountable for all aspects of the work in ensuring that questions related to the accuracy or integrity of any part of the work are appropriately investigated and resolved.

### Conflict of Interest

The authors declare that the research was conducted in the absence of any commercial or financial relationships that could be construed as a potential conflict of interest.

## Footnotes

^1^The Israeli compulsory education law. Available online at: http://www.google.com/url?sa=t&rct=j&q=&esrc=s&source=web&cd=5&ved=2ahUKEwip0eeBp8PkAhUFa1AKHabqBE0QFjAEegQIAxAC&url=http%3A%2F%2Fedu.gov.il%2FDocuments%2Flimudchova.doc&usg=AOvVaw0fn3bowWd59V_cDnVqL4lB.
